# A qualitative study of the stress autism mate app among autistic adults: user experiences and effects on stress awareness and coping skills

**DOI:** 10.3389/fpsyt.2025.1637008

**Published:** 2025-08-21

**Authors:** Kirsten Hoeberichts, Yvette Roke, Frank Damen, Irene Niks, Peter N. Van Harten

**Affiliations:** ^1^ Expertise Centre Specialised in Autism Spectrum Disorder, GGz Centraal, Almere, Netherlands; ^2^ Department of Psychiatry and Neuropsychology, School of Mental Health and Neuroscience, Maastricht University, Maastricht, Netherlands; ^3^ Department of Psychiatry, GGz Centraal, Amersfoort, Netherlands; ^4^ Department Work, Health and Technology, The Netherlands Organization for Applied Scientific Research (TNO), Leiden, Netherlands

**Keywords:** autism spectrum disorder, stress management, mobile health (mhealth), autistic adults, coping strategies, qualitative research, self-regulation, digital mental health intervention

## Abstract

**Introduction:**

Autistic adults often face unique challenges in stress management. Conventional tools may not cater to their distinct needs. The Stress Autism Mate (SAM) app was developed to support stress recognition and promote active coping strategies through structured self-monitoring and personalised feedback. This study explored how autistic outpatient adolescents and adults experience the use of SAM in relation to stress awareness, coping behaviours, and engagement with digital tools.

**Methods:**

A qualitative phenomenological design was used, involving in-depth interviews with ten autistic participants (N = 10) who used SAM for at least four weeks. Reflexive thematic analysis was conducted to identify key patterns in user experiences.

**Results:**

Three key processes were identified: (1) SAM facilitated increased awareness of previously unrecognised stress by externalising internal states, (2) participants shifted from avoidant to active coping strategies, supported by structured reflection and coping suggestions, and (3) the app’s emotionally neutral, predictable design created a safe and engaging space for self-regulation. However, tensions between structure and flexibility highlighted the need for greater personalisation to sustain engagement over time.

**Discussion:**

In conclusion, SAM supports autistic individuals in transforming vague stress experiences into actionable insights, fostering emotional literacy and coping capacity. These findings extend prior quantitative evidence on SAM’s efficacy and provide actionable design recommendations for mHealth interventions aimed at neurodivergent populations.

## 1 Introduction

Autism spectrum disorder (ASD) is a lifelong developmental disorder with an estimated prevalence of 1% worldwide (1–3). ASD is characterised by a spectrum of core symptoms that vary in severity and manifest within two domains: (1) impairments in social communication and (2) restricted and rigid patterns of interests or activities (4, 5). Autistic adults process stimuli and information in a manner that differs from that of adults without autism. This can result in difficulties in integrating detailed information into a coherent whole. Such difficulties can give rise to problems with communication and social interaction (6, 7). Furthermore, autistic adults frequently experience elevated levels of stress compared to adults without autism (8). These elevated stress levels can further complicate daily functioning, thereby underscoring the importance of effective and active coping strategies for stress reduction of autistic adults (6, 8, 9).

To manage stress, autistic adults frequently employ passive coping strategies, such as avoiding social situations and focusing on emotions like anger. However, these maladaptive coping strategies have the potential to intensify stress (6, 8, 10, 11). In this study stress is defined as a psychological state in which an individual perceives that the demands of a situation exceed their available coping resources, particularly in unpredictable or uncontrollable contexts (12, 13). Manageable levels of stress can act as a motivating force, helping individuals to effectively tackle challenges. In contrast, chronic stress arises when stressors persist and exceeds an individual’s ability to cope. Chronic stress is linked to a range of adverse physical and mental health outcomes, including hypertension, heart palpitations, and an increased vulnerability to depression and anxiety disorders (14, 15). Over time, chronic stress can lead to a number of adverse effects, including poorer clinical results, reduced treatment efficacy, diminished resilience, and a lower overall quality of life (9, 12, 16, 17).

The current treatment of autistic adults with a normal IQ is based on psychoeducation, counselling in the psychosocial area and the treatment of any comorbid problems (18–20). One element of the treatment plan is the formulation of a stress-signalling plan, which delineates the stress signals and corresponding actions to be taken in order to prevent relapse. It is essential that the individual using this signalling plan is able to accurately identify the onset of stress. This can prove challenging for autistic adults, due to alexithymia and their unique information-processing abilities (21, 22a). In everyday situations, stress is often unnoticed and not recognised in a timely manner. Consequently, a stress-signalling plan is not the most appropriate intervention for this population. Autistic adults require a more appropriate tool to facilitate the learning of stress recognition.

To address this gap, the Stress Autism Mate (SAM) app was developed by XXXX. The SAM app was developed in cocreation with end users, namely autistic adults, with the objective of creating an intuitive and responsive app that caters to the user’s needs regarding the use of language, colouring schemes and the touch and feel of the app. This was achieved through the use of the design thinking method (23, 24) which was employed in the development of SAM, thereby ensuring a user-centred approach. SAM is a personalised app designed for autistic adults with a normal IQ (>85). Its objective is to assist users in recognising their perceived stress levels, gaining insight into their stress patterns, and reducing stress through the use of personalised stress tips provided by SAM. Consequently, users are able to learn to manage their stress more effectively. SAM is available for free in seven languages in the App Store and Google Play in Europe, New Zealand, Australia, and Canada^1^.

Previous quantitative research on the effectiveness of the SAM app in autistic adults is emerging. To date, three quantitative studies have been conducted with autistic participants: one pre-post study N = 15 (25), one single case experimental design study N = 36 (26) and one recent randomized controlled trial with 87 participants (27). These studies consistently reported that four weeks of SAM use led to reductions in perceived stress, improved coping skills, and greater satisfaction with health, self-stigma, and resilience, with effects sustained at one-month follow-up. While these findings are promising, they rely exclusively on quantitative methods and therefore provide only a partial picture. To fully understand the impact of SAM, it is essential to explore the subjective, lived experiences of its users. The present study addresses this gap by adopting a qualitative approach, offering deeper insights into how autistic adults engage with SAM in their daily lives.

Gaining this qualitative perspective is not only important for contextualizing and enriching previous findings, but also for informing the optimization of SAM itself. Understanding how autistic adolescents and adults experience the app in relation to stress awareness and coping behaviours can guide improvements to its design and functionality. However, more importantly, it contributes to our understanding of how mHealth interventions can be better designed and implemented to meet the specific needs of individuals with autism. To date, no qualitative research has been conducted on the experiences of SAM users. As a result, the manner in which SAM influences their coping skills and overall experience remains unclear.

This study focuses on understanding how autistic adolescents and adults (aged 16 and above) experience the use of the SAM app, with particular attention to its role in promoting stress awareness and influencing coping behaviours. By exploring these subjective experiences, the research aims to generate user-centred insights that can inform the design and development of mHealth tools better suited to the needs of individuals with autism. To this end, the following research question was posed: ‘How do autistic adolescents and adults experience using the SAM app in relation to stress awareness and coping strategies’?

## 2 Materials and methods

### 2.1 Study design

This qualitative study employed a reflexive thematic analysis (28, 29) to explore the lived experiences of autistic outpatient adolescents and adults using the SAM app. This approach enabled an in-depth examination of how users engaged with the app in relation to stress recognition and coping behaviours, generating insights to inform future mHealth design.

### 2.2 Participants

The study was conducted through in-depth interviews with men and women over the age of 16 who had been diagnosed with ASD. The participants were undergoing treatment at XXXX an academic centre for child and adolescent psychiatry. Participants aged 16 and older were included because the participating clinical centres provide treatment for both adolescents and adults with autism. Including a broad age range allowed us to capture diverse perspectives on the usability and impact of the SAM app across different stages of life. While this introduces some heterogeneity, it reflects the real-world diversity of app users and supports the exploration of how individual differences shape stress management experiences. The clinicians at these institutions selected participants from their caseloads based on the inclusion criteria: (1) a formal diagnosis of Autism Spectrum Disorder according to DSM-5 or ICD-10, (2) aged 16 years or older, (3) having an IQ equal or higher than 85 according to the Wechsler Adult Intelligence Scale IV Dutch (WAIS-IV-NL) (4) treatment at XXXX, (5) having used the SAM app for a minimum of four weeks, and (6) the ability to reflect on their experiences with SAM and its impact on their coping skills. A total of 11 participants were initially enrolled in the study. Two of the participants were undergoing treatment at XXXX, while the remaining nine were undergoing treatment at XXXX. Eleven individuals were initially enrolled in the study. One individual was excluded due to a lack of response to repeated invitations, resulting in ten participants being interviewed. Among them, six (60%) were women and four (40%) were men, aged between 17 and 63 years. **Table 1** provides an overview of the demographic characteristics of the participants.

**Table 1:** Demographic and baseline characteristics of study participants sorted by age (N=10).

Respondent number	Gender	Age	Diagnosed in	Treatment since
1	♀	58	2012	2012; 2021
2	♀	19	2021	2015
3	♂	63	2020	2021
4	♂	19	2007	2021
5	♀	17	2016	2012
6	♀	62	2018	2018 t/m 2021
7	♂	58	2019	2018
8	♀	22	2006, 2018	2018
9	♀	32	2021	2021
10	♂	33	2011	2011; 2021

♀, female; ♂, male.

The respondent numbers correspond with the coding used in the text. To illustrate: “feeling restless in my head and irritable” (R1) is a quote from participant 1.

### 2.3 Intervention

**Figure 1:**
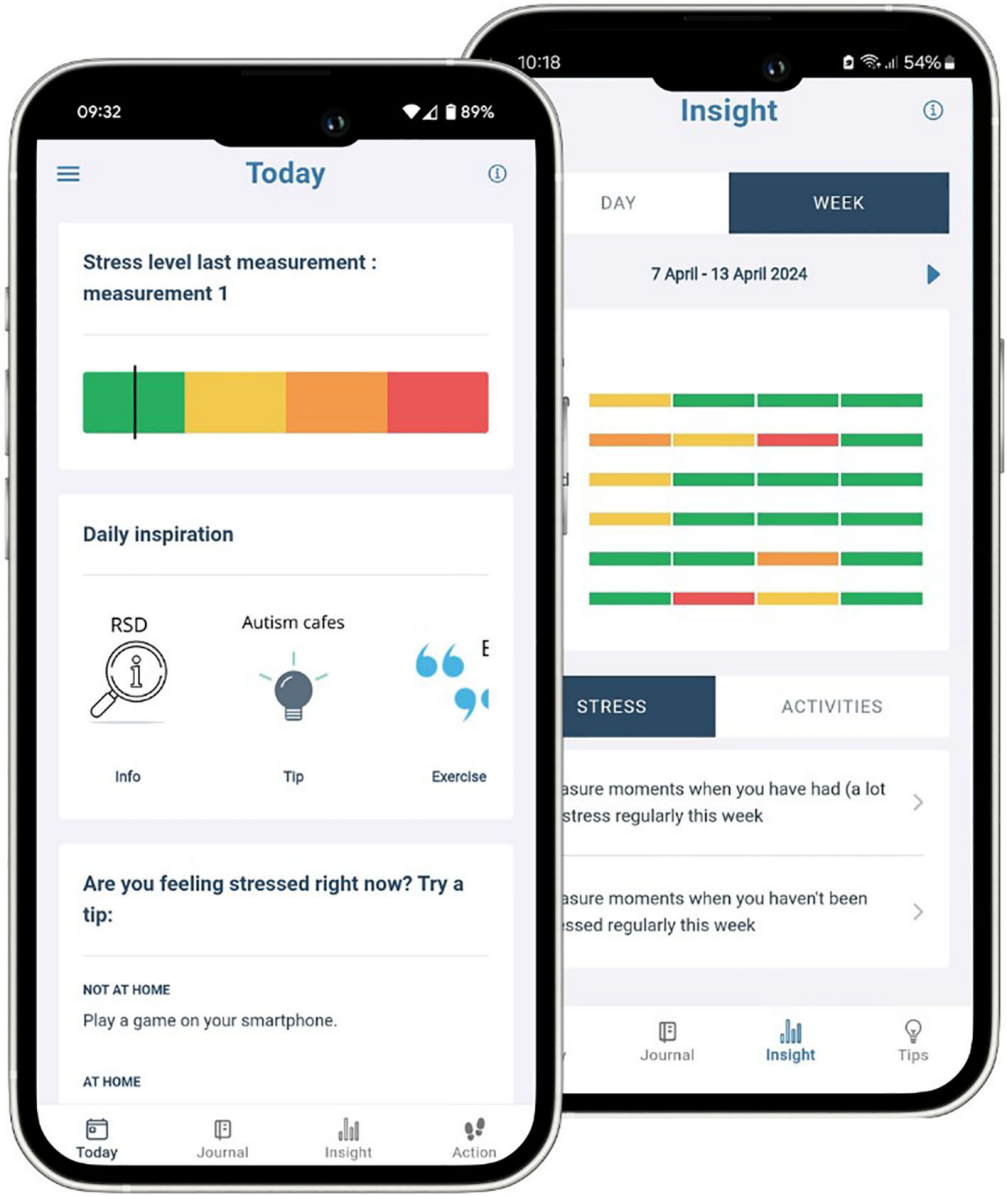
Screenshots of the SAM app’s “Today” page and “Stress Insight” graph, illustrating daily self-assessment and stress tracking features.

SAM is a self-help mobile application designed to assist users in managing and understanding daily stress. The app was developed in co-creation with end-users. Their feedback was incorporated into its design and features. The app prompts users to complete a questionnaire four times a day, focusing on their activities and stress levels over the past four hours. SAM employs a research-driven algorithm to evaluate the responses provided by the user and determine a stress level. Subsequently, the user is prompted to review the level to ensure its accuracy. The app offers personalised coping strategies to assist users in mitigating stress that has been identified. Furthermore, SAM provides users with visual representations of their stress levels, which are presented in the form of graphs that illustrate both daily and weekly trends (**Figure 1**). Further comprehensive information about SAM’s features and development can be found in a prior publication (25).

### 2.4 Data collection

The interviews were conducted between November 2021 and March 2022, with informed consent obtained in advance. Six interviews were conducted via digital platforms (Microsoft Teams), while the remaining four were conducted on-site at XXXX. The duration of each interview was approximately 30–60 minutes, and it was audio-recorded with the participants’ consent.

### 2.5 Data analysis

The interviews were transcribed in their entirety and subjected to thematic analysis in accordance with the six-step approach outlined by Braun and Clarke (28). The following stages were undertaken: familiarisation with the data, generation of initial codes, search for themes, review of themes, definition and naming of themes, and production of the report. The data were analysed using the Atlas.ti software package. The most pertinent statements were selected and assigned an open code, which reflected the core essence of the statement. Subsequently, the corresponding codes were merged into code groups. The analysis of these code groups and the underlying data led to the identification of themes. Memo writing was conducted after each interview to capture initial impressions and support reflexive engagement with the data. Themes were developed and refined collaboratively by two researchers to enhance interpretive depth and rigor. Throughout the analysis, a reflexive and interpretative stance was maintained, acknowledging the researchers’ active role in constructing meaning from participants’ accounts. The point of saturation was reached after ten interviews, as evidenced by the emergence of consistent patterns and the absence of new information.

### 2.6 Ethical considerations

The Medical Ethical Review Committee (METC) of Isala in Zwolle issued a non-WMO statement under number 210717. Prior to their involvement in the study, participants were provided with comprehensive details regarding the nature of the study and were asked to provide written informed consent. The confidentiality of the data was guaranteed through the anonymisation of the transcripts and the secure storage of the data.

### 2.7 Trustworthiness

To ensure the methodological quality of the study, the criteria of reliability (trustworthiness) were applied, which strengthened the credibility, transferability, dependence, persuasiveness and authenticity of the study (30, 31). Data analysis and coding were conducted thoroughly by both the interviewer and a peer-reviewer. The insights gained were used in follow-up interviews. The results section includes extensive descriptions of quotes from participants, which enhanced the authenticity of the data. Furthermore, participants were given the opportunity to read and identify with the research findings, thereby further strengthening the credibility and persuasiveness of the study.

### 2.8 Community involvement

Apart from the co-creation process of SAM with adults with autism, there was no public community involvement in this research.

## 3 Results

### 3.1 From unnoticed to understood: recognising stress

Prior to SAM, participants reported experiencing daily stress. However, they were unable to recognise the early signs of low stress levels or the gradual accumulation of stress, which resulted in a, for the user ‘sudden,’ rapid increase in stress levels resulting in physical or emotional complaints. Stress was described as “feeling restless in my head (R1)”, and “a full or busy head” (R5) or “never having rest (R3, R7)”, yet few participants were able to identify specific triggers or early warning signs. Instead, they found themselves “always going on and on” (R1), “never having rest” (R3, R7) or “not noticing that I am running out” (R5). This pattern reflects a broader challenge in stress perception and self-awareness. For many participants, stress existed as a diffuse, embodied experience that lacked clear emotional or cognitive markers. These difficulties align with characteristics commonly found in individuals with autism, such as alexithymia or impaired interoceptive awareness, where internal states are difficult to access or articulate (21, 22, 32).

Participants described SAM as a helpful tool that introduces structure and supports greater awareness of otherwise unrecognized stress. Participants described SAM’s role in “forcing me to think” (R1, R6) and in “putting words to my feelings” (R2). Over time, they reported greater insight not only into their stress levels but also into their behavioural patterns, saying, for instance, “I realized I experienced more daily stress than I thought” (R4) and “I now understand what causes my stress” (R6). This growing self-awareness laid the foundation for more effective regulation and coping.

### 3.2 Building resilience: from passive to active coping

A key transformation observed in participants was a shift from passive or avoidant coping strategies toward more active and intentional forms of stress management (R2, R5, R9). Prior to using SAM, many described reacting to stress through withdrawal, irritability, or emotional outbursts: “I would lash out” (R7) or “flee the situation” (R3, R7). Furthermore, there were also coping behaviours that were detrimental to the individual’s well-being, including drug use (R4, R9). These responses often reinforced the stress cycle, leaving participants feeling powerless or disconnected.

While using SAM, users began to develop a broader coping repertoire. The app’s task-oriented tips—such as engaging in distraction, relaxation exercises, or seeking support—helped initiate behavioural change. Participants described these tips as “a useful reminder” (R2), “SAM has given me insight into how to do things differently” (R4), and several reported successful applications in real time: “My head gets calmer, the stress is gone” (R1).

Over time, users adopted four primary forms of active coping:

1. Problem-focused coping – planning and adjusting behaviour to reduce stress (e.g., “My stress doesn’t get so overwhelming. I manage my appointments better” R6),
2. Cognitive coping – reframing thoughts and offering self-reassurance (e.g., “I reassure myself in situations” R6),
3. Distraction and relaxation – using calming activities to regulate arousal (e.g., “I sit with my dog and feel better” R10),
4. Seeking social support – reaching out to others (e.g., “I email someone or contact them when I don’t feel well” (R 9).

This shift in coping style marked not only a behavioural change but also an increase in self-efficacy and emotional literacy. Two participants even reported discontinuing the use of SAM, stating they had internalised the skills and no longer required external support (R5, R6). Conversely, two others felt the app did not meet their specific needs (R3, R10), suggesting that while SAM supports users, it may require further personalisation to remain effective for all.

While these coping strategies evolved over time, participants frequently attributed this shift to the structured and consistent nature of the SAM app. The next theme explores how SAM’s design and features contributed to this transformation.

### 3.3 Structured support: how technology supports self-regulation

SAM was widely perceived as more than just a monitoring tool—it acted as a cognitive framework that supported self-regulation, provided predictability, and promoted reflection. By prompting users four times a day with a set of structured questions, SAM imposed a rhythm that was appreciated: “It gives me discipline” (R4, R9) and “It helps me reflect on my day” (R3). Participants highlighted the value of these recurring check-ins as “enforced moments of reflection” (R3), making stress a tangible, nameable experience.

The feedback SAM provided—especially through colour-coded diagrams—served as a visual representation of emotional states. Many participants referred to these visual aids as a “stress gauge” (R5) or a “statistic of the past week” (R2), which helped them understand their stress patterns over time. In this sense, SAM was experienced as a form of external support that helped some users observe, interpret, and reflect on their internal states with greater clarity.

Importantly, participants viewed SAM as emotionally neutral and non-judgmental, describing it as “clear and honest” (R5), which made its suggestions more acceptable than those from “someone with emotion” (R5, R9). This emotional distance was perceived as helpful, with one participant noting, “It’s easier when something just tells me what to do” (R9). Beyond neutrality, some participants also ascribed a sense of companionship and nuance to the app, referring to SAM as “a buddy” (R1) and “a helpful tool that shows not only black and white but also shades of grey” (R7). These descriptions suggest that SAM was experienced not only as a source of structure and feedback, but also as a non-intrusive, supportive presence in participants’ daily lives.

However, SAM’s structured design was not without limitations. Some participants felt overwhelmed by the frequency of prompts, suggesting “three times a day would also be enough” (R1, R9), this resulted in the non-completion of some questionnaires (R3, R10). Others noted frustration with the rigid 60-minute response window and weekly password logins, which were described as stress-inducing rather than supportive (R2, R3, R6). The repetition of tips and lack of personalisation sometimes led to disengagement, and some found the interface “impersonal” or wished it addressed them by name (R7). These reactions reveal a tension between the benefits of structure and the potential rigidity of digital tools.

## 4 Discussion

This study explored the lived experiences of outpatient autistic adolescents and adults using the SAM app to support stress recognition and management. The analysis revealed a three-fold process in participants’ experiences of this study: (1) increased stress awareness, (2) the development of active coping strategies, and (3) the role of SAM’s digital design. These are deeply interconnected. Enhanced emotional awareness, fostered by the app’s structured prompts and visual feedback, lays the groundwork for users to engage in more effective coping behaviours. In turn, the app’s design elements, such as its predictability and emotional neutrality, both support and sometimes challenge this process, highlighting important considerations for user engagement and personalization. These qualitative results extend previous quantitative findings (25, 26), which demonstrated reductions in perceived stress and increases in coping ability and resilience. Together, these findings offer a cohesive framework for understanding how mHealth tools like SAM can facilitate emotional self-regulation and stress management in autistic individuals. And thereby the data contributes to the growing evidence base supporting the use of mHealth tools for autistic individuals (33–35). However, participants also identified limitations related to the app’s rigidity and repetition, underscoring the need for greater personalisation and flexibility in digital mental health interventions.

### 4.1 Understanding stress through externalised reflection

A central mechanism through which SAM influenced participants’ experiences was its ability to externalise internal emotional states. Prior to using the app, many participants described stress as an indistinct, cumulative sensation lacking specific cognitive or emotional markers. This challenge is consistent with established findings on alexithymia and interoceptive difficulties commonly observed in autistic individuals (36), both of which impair emotional awareness and can lead to delayed or avoidant responses to stress (21, 22, 32). Such impairments often result in reactive behaviours that reinforce emotional dysregulation (6, 37, 38). Within this context, SAM functioned as a mediating tool by offering a structured and predictable framework for emotional self-assessment. Through daily check-in prompts and the colour-coded diagrams, the app supported users in identifying and interpreting their internal states with greater clarity.

Participants’ favourable responses to SAM’s visual design and structured prompts reflect a broader preference among many autistic individuals for routine, predictability, and low-verbal cognitive processing (39, 40). The combination of specific, stress-focused questions and colour-coded diagrams enabled users to translate vague somatic or emotional cues into more concrete representations. This process aided both verbalisation and emotional insight, with several users referring to the app’s feedback as a “stress gauge” or “weekly statistics,” highlighting its role as a visual anchor. In this way, SAM contributed to making stress “visible,” facilitating a clearer understanding of emotional fluctuations and triggers over time. This mechanism aligns with previous research showing that digital tools can serve as compensatory strategies for individuals with difficulties in self-monitoring and executive functioning (23). Furthermore, the routine of daily self-reflection appeared to cultivate a habit of checking in with one’s emotional state—an effect supported by earlier work on ecological momentary assessment and stress-tracking tools (41–43). For participants in this study, the repetition and structure embedded within SAM helped to develop a reflective practice that gradually enhanced awareness and recognition of stress.

### 4.2 Developing coping capacity: from avoidance to action

While increased awareness and recognition of stress are essential steps, it is the translation of insight into action that ultimately leads to behavioural change. Participants described a shift from avoidant or reactive coping strategies—such as emotional outbursts, withdrawal, or substance use—to more active forms of stress management. These included problem-solving, cognitive reframing, distraction techniques, and seeking support. By providing concrete, repeatable, and easily accessible stress-reduction tips, SAM contributed to the expansion of users’ coping repertoires.

This transition mirrors Lazarus and Folkman’s (44) cognitive coping model, in which the evaluation of the situation precedes the selection of coping strategies (45). SAM facilitated this appraisal process by providing structured reflection, while the readily available stress-reduction tips — despite occasional repetition — encouraged direct adaption of coping strategies. The observation that some users internalised these strategies over time and continued to apply them even after discontinuing the app suggests that SAM may act as an intermediate step in the transition from supported learning to autonomous self-regulation.

**Figure 2:**
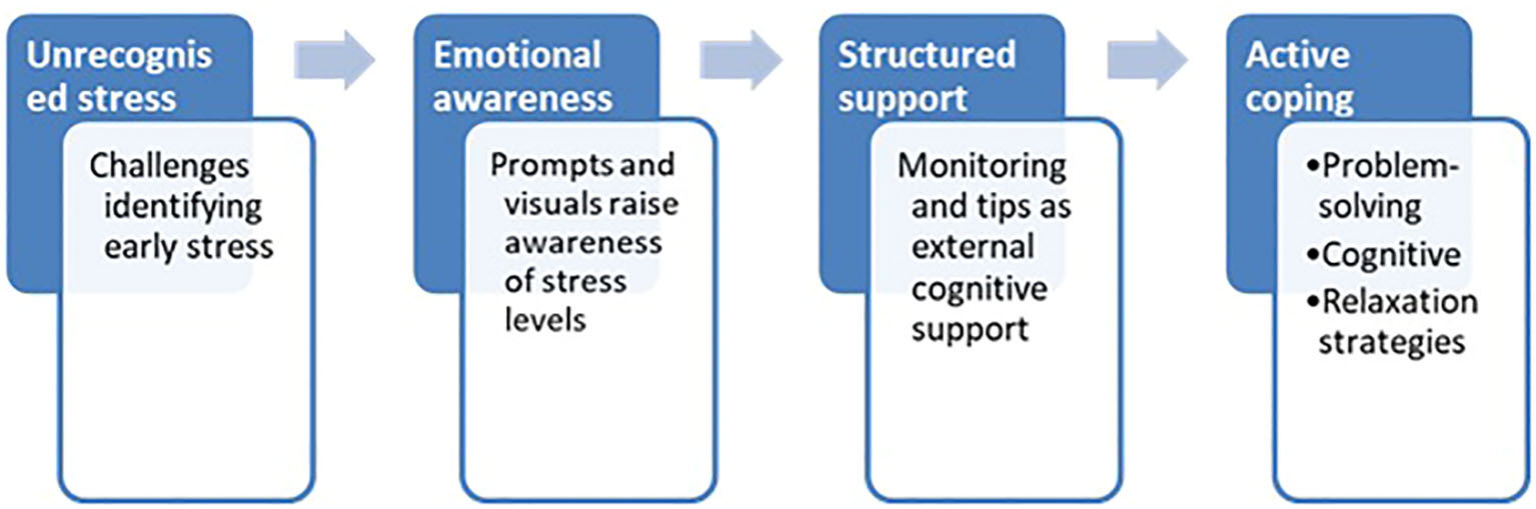
Conceptual model of stress awareness and coping development facilitated by the SAM app. The model visualises four functional shifts described by participants: from unrecognised stress to emotional awareness, structured support, and ultimately active coping. Each step is supported by a distinct role of SAM—such as externalising internal states, prompting reflection, and reinforcing behavioural strategies. Rather than a fixed sequence, the model represents recurring turning points in users’ engagement with the app, offering insight into how digital tools may scaffold self-regulation in autistic individuals.

This process may also reflect the principle of “coping skill acquisition” observed in other therapeutic contexts, where structured prompts and immediate feedback help reinforce adaptive behaviour (46). Similarly, Hoeberichts et al. (25, 26)) reported increased coping confidence among users following regular engagement with SAM. These findings are synthesised in **Figure 2**, which offers a conceptual lens on the developmental nature of participants’ coping experiences. Rather than fixed stages, each phase reflects a functional shift supported by SAM—ranging from externalising emotional states to reinforcing coping strategies. These shifts were not uniform or strictly linear; instead, they represent recurring turning points where users re-engaged with the app to regulate their internal states. By framing the findings in this way, the model clarifies the mechanisms through which structured, emotionally neutral tools like SAM can facilitate emotional awareness and behavioural adaptation. It may also serve as a guiding framework for the design of future mHealth interventions aimed at supporting self-regulation in neurodivergent populations.

### 4.3 Digital design and the value of emotional neutrality

A notable aspect of SAM’s effectiveness lies in its balance between emotional neutrality and structured interaction. Participants consistently valued the app’s predictable, non-judgmental interface and clear feedback, describing it as “honest,” “a buddy,” and “a helpful tool that shows not only black and white but also shades of grey.” These qualities and SAM features, including clear visual feedback and emotionally neutral language appeared to enhance receptivity to feedback, particularly when compared to human interactions, which were often experienced as emotionally ambiguous or overwhelming (47).

However, while many found SAM helpful and supportive, some users expressed frustration or disengagement due to the app’s rigidity; specifically its fixed timing of prompts, repetitive suggestions, and limited personalisation. This diversity of user experiences highlights the tension between the value of consistency and the need for adaptability, a challenge well recognised in mHealth research where overly rigid systems can undermine long-term adherence and responsiveness to evolving needs (48, 49). The current findings contribute to this discourse by illustrating how even subtle inflexibilities in app design can affect user engagement, particularly in neurodivergent populations where sensory sensitivities and fluctuating capacities for engagement are common.

These insights underscore an important design principle for future iterations of SAM and similar digital interventions: the need to maintain a balance between structure and personalisation. Participants’ suggestions—such as adjustable prompt frequency, greater variety in coping strategies, and a more tailored interface—echo ongoing developments within the SAM project (26, 50). Embedding greater user-configurable features may not only enhance usability but also respect the autonomy and heterogeneity of autistic users, ultimately strengthening both engagement and clinical effectiveness.

### 4.4 Implications for mHealth design and clinical practice

This study makes a significant contribution to the expanding domain of digital mental health tools by demonstrating the efficacy of structured, emotionally neutral tools in supporting self-regulation among autistic individuals. While SAM has been developed for autistic individuals, the mechanisms it employs — namely, the externalisation of stress, introduction of reflection routines, and offering actionable tips — are likely to be relevant across a range of neurodivergent or high-stress populations.

The SAM app exemplifies how digital interventions can serve as low threshold complements to traditional care. In clinical practice, SAM could be integrated into individualised stress-signalling plans or offered as a daily tool to support reflection between sessions. Given its potential to promote self-awareness, agency, and behavioural change, SAM may also help reduce reliance on in-person care—especially important in contexts of limited access or long waiting lists. Notably, the app is freely available and accessible in multiple languages, which increases its reach and cost-effectiveness. These features may make it a suitable option for wider implementation, including in early-stage support or monitoring settings, although further research is needed to explore its preventive potential.

### 4.5 Limitations

While this study provides valuable insights, several limitations must be acknowledged. First, the sample size was relatively small. However, in qualitative research, sample adequacy is determined by data saturation rather than numerical thresholds. In this study, data saturation was achieved after ten interviews, suggesting that key themes were thoroughly explored.

Secondly, participants were recruited from specialised outpatient autism centres, which may limit the generalisability of the findings to the broader autistic population. These services often support individuals with more complex needs, potentially making the sample less representative of those in general care. Nevertheless, the positive outcomes reported by this group suggest that the SAM app may be equally or even more effective in less complex cases. As a low-threshold, free, and immediately accessible tool, SAM may hold particular value for individuals with limited access to professional mental health services.

Thirdly, while participants were required to have an IQ of 85 or higher, the study did not formally assess verbal, cognitive, or executive functioning abilities. Given that the SAM app relies on self-reflection, written responses, and consistent engagement, variations in these cognitive and communication skills may influence user interaction and outcomes. This limitation should be considered when interpreting the transferability of findings to autistic individuals with more diverse cognitive profiles. Future research could explore the app’s applicability and usability across a broader range of cognitive and communication abilities.

Fourthly, the use of self-reported data introduces the possibility of response bias or social desirability, whereby participants may have provided answers that are perceived as socially desirable or may not have recalled their experiences with complete accuracy. However, self-reported data is a widely accepted and valid method in qualitative research, particularly when exploring personal experiences and perceptions. The use of open-ended questions and subsequent probing in the interviews served to mitigate the risks associated with response bias by encouraging participants to reflect deeply and provide more considered responses.

Furthermore, the qualitative study design does not allow for the examination of long-term effects associated with SAM use. While this design limits the ability to assess changes over time, it does provide a representative sample of participants’ current experiences and perceptions, which are valuable for understanding the immediate impact of the app.

### 4.6 Future directions

The potential of SAM extends beyond autistic individuals, suggesting that it could be beneficial for a broader audience, including those with various psychological and somatic conditions. Stress is a common factor in both mental and physical health issues and can exacerbate symptoms (51). For instance, SAM Junior, an app designed for autistic adolescents aged 12-18, exemplifies how SAM’s core functionalities can be adapted for different age groups and needs (50) (https://samjunior.nl). Further research is recommended to explore SAM’s application across wider target groups. This broader applicability could lead to more integrative and accessible stress management approaches in clinical practice, enhancing overall patient care across diverse conditions.

The ongoing development of the app is significantly influenced by the active SAM panel, which comprises over three hundred Dutch autistic members. The suggestions put forth by the SAM panel are subject to continuous review and integration, thereby ensuring that SAM evolves in response to user feedback. This iterative process includes regular updates and validations by the panel, which serve to illustrate the vital role they play in maintaining the app’s effectiveness and relevance. Furthermore, the proposed enhancements suggested by the participants of this study will be incorporated into forthcoming SAM updates and other prospective applications, thereby ensuring uninterrupted advancement and adaptability.

## 5 Conclusion

This qualitative study offers insight into the mechanisms through which the SAM app supports stress awareness and coping in autistic adolescents and adults. By helping users externalise and reflect on emotional states, SAM facilitated a shift from passive to active coping strategies and was generally experienced as a predictable, non-intrusive tool. While the app’s structured nature was key to its value, future iterations must balance consistency with flexibility to meet diverse user needs. These findings extend previous quantitative evidence (25, 26) and provide actionable insights for designing digital interventions that foster self-regulation and emotional resilience in neurodivergent populations. You may insert up to 5 heading levels into your manuscript as can be seen in “Styles” tab of this template. These formatting styles are meant as a guide, as long as the heading levels are clear, Frontiers style will be applied during typesetting.

## Data Availability

The raw data supporting the conclusions of this article will be made available by the authors, without undue reservation.

## Footnotes

1 SAM is freely available for download from the Google Play Store and the Apple App Store in 33 countries (see Appendix A) and in multiple languages, including Dutch, English, German, Ukrainian, Turkish, Danish, and French. For more information, visit https://www.stressautismmate.nl/.
